# The role of α-hydroxybutyrate in modulating sepsis progression: identification of key targets and biomarkers through multi-database data mining, machine learning, and unsupervised clustering

**DOI:** 10.3389/fphar.2025.1615269

**Published:** 2025-09-17

**Authors:** Qing Lu, Yujie Wu, Dayong Liao, Ying Sun

**Affiliations:** ^1^ Department of Geriatrics, Sichuan Provincial People’s Hospital, University of Electronic Science and Technology, Chengdu, China; ^2^ Institute of Geriatric Diseases, Sichuan Provincial People’s Hospital, University of Electronic Science and Technology, Chengdu, China; ^3^ School of Medicine, University of Electronic Science and Technology, Chengdu, China

**Keywords:** gut microbial metabolites, sepsis, machine learning, immune response, molecular docking, personalized medicine, bioinformatics

## Abstract

**Background:**

Sepsis remains a major cause of mortality and morbidity worldwide. Recent studies suggest that gut microbiota-derived metabolites, such as α-hydroxybutyrate (α-HB), may play a critical role in the progression of sepsis. However, the molecular mechanisms underlying α-HB’s involvement in sepsis remain unclear. This study aims to explore the targets of α-HB and their association with sepsis progression using multi-database data mining, machine learning, and unsupervised clustering analyses.

**Methods:**

α-HB-related targets were identified through comprehensive data mining from three databases: SEA, SuperPred, and SwissTargetPrediction. Sepsis-related targets were obtained from the GEO dataset GSE26440, and the intersection of these datasets was analyzed to reveal common targets. Functional enrichment analysis, protein-protein interaction (PPI) network construction, and machine learning algorithms (L1-LASSO, RF, and SVM) were applied to identify biomarkers. Additionally, a nomogram was constructed to predict sepsis progression. Clustering, GSVA, and ssGSEA analyses were performed to explore sepsis subtypes. Molecular docking simulations was conducted to investigate interactions between α-HB and key targets.

**Results:**

A total of 42 common targets were identified between α-HB and sepsis, with significant enrichment in pathways related to immune response, hypoxia, and cancer. Machine learning-based feature selection identified four robust biomarkers (APEX1, CTSD, SLC40A1, PIK3CB) associated with sepsis. The constructed nomogram demonstrated high predictive accuracy for sepsis risk. Unsupervised clustering revealed two distinct α-HB-related sepsis subtypes with differential immune cell infiltration patterns and pathway activities, particularly involving immune and inflammatory pathways. Subtype 1 was predominantly associated with non-survivors, while Subtype 2 was more frequent among survivors, showing a significant difference in survival status. Molecular docking analysis further indicated potential interactions between α-HB and key targets (APEX1, CTSD, SLC40A1, PIK3CB), providing insights into the molecular mechanisms of α-HB in sepsis.

**Conclusion:**

This study identifies key α-HB-related targets and biomarkers for sepsis, offering new insights into its pathophysiology. The findings highlight the potential of α-HB in modulating immune responses and suggest that α-HB-related targets could serve as promising therapeutic targets for sepsis management.

## Introduction

Sepsis continues to be a prominent cause of morbidity and mortality globally, defined by a disordered immune response to infection that gives rise to systemic inflammation, multiple organ dysfunction, and frequently leads to death (Cecconi et al., 2018; Ackerman et al., 2021). Despite substantial advancements in clinical management, the early diagnosis and effective treatment strategies for sepsis remain highly challenging (Gauer et al., 2020; Rello et al., 2017). Recent studies have placed emphasis on the significance of the gut microbiota and its metabolites in regulating host immune responses, influencing the onset and advancement of sepsis (Ma et al., 2023; Kullberg et al., 2021). For example, it was discovered that the gut-derived metabolite rhamnose functions as a molecular agent that enhances the phagocytic ability of macrophages, thereby offering protection to the host in cases of sepsis (Li et al., 2024). Additionally, the gut microbial metabolite hyodeoxycholic acid interacts with the TLR4/MD2 complex, leading to a reduction in inflammation and providing defense against sepsis (Li et al., 2023). Among these metabolites, α-hydroxybutyrate (α-HB), a short-chain organic acid produced through both gut microbial metabolism and amino acid catabolism and associated with oxidative stress, has emerged as a potential crucial player in modulating inflammatory and immune responses (Lv et al., 2023). α-HB demonstrates a significant correlation with the initiation of sepsis as well as the mortality rate observed at 28 days (Zhao et al., 2023). However, comprehensive data regarding its physiological concentration ranges and specific changes in systemic levels during sepsis remain limited in the current literature. Furthermore, the molecular mechanisms underlying α-HB’s influence on sepsis progression remain poorly understood, and its precise therapeutic potential and pathophysiological significance in this context require further clarification.

α-HB has been shown to exert multiple biological effects, including modulation of immune responses, regulation of cellular metabolism, and the maintenance of metabolic homeostasis. Notably, the increased levels of α-HB may represent a new common risk factor associated with the correlation between colorectal cancer and diabetes (Lv et al., 2023; Rafaqat et al., 2024). Emerging evidence suggests that metabolites like α-HB could play pivotal roles in diseases with complex inflammatory pathways, such as sepsis (Zhao et al., 2023). However, despite its potential, the relationship between α-HB and sepsis is not well-characterized, and the molecular targets through which α-HB may influence sepsis progression remain largely undefined. This lack of understanding underscores the need for comprehensive studies that integrate multiple bioinformatics approaches to identify potential molecular pathways and biomarkers that could inform both early diagnosis and therapeutic strategies.

**FIGURE 1:**
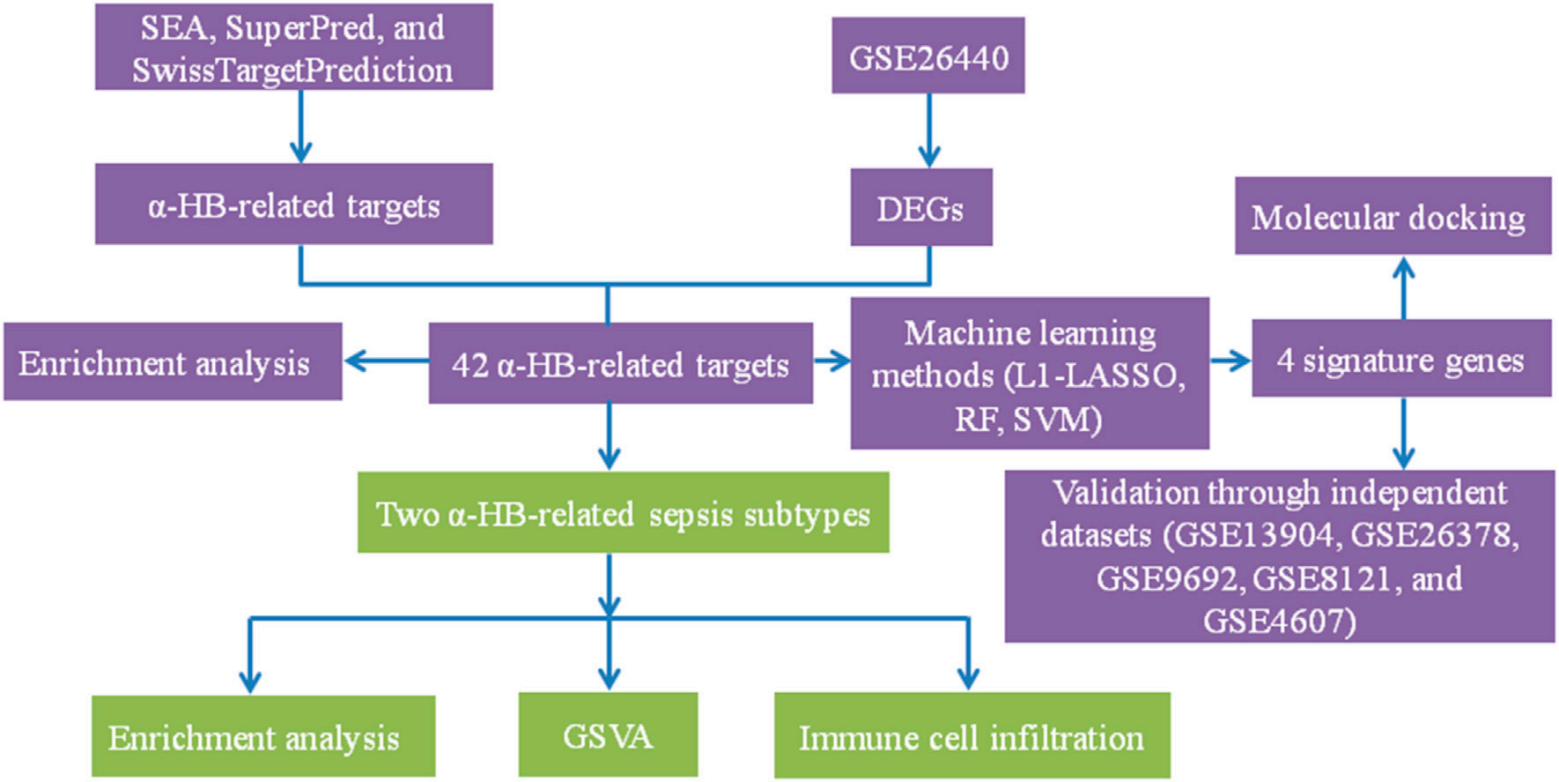
The detailed workflow of the study. This flow chart illustrates the comprehensive steps undertaken in the study, including the identification of α-HB-related targets through SEA, SuperPred, and SwissTargetPrediction databases. Sepsis-related DEGs were obtained from the GSE26440 dataset. Machine learning methods (L1-LASSO, RF, SVM) were then employed to identify four signature genes. These genes were validated through independent datasets and analyzed via molecular docking. Unsupervised clustering revealed two distinct α-HB-related sepsis subtypes, which were further characterized using enrichment analysis, GSVA, and immune cell infiltration analysis.

Therefore, in this study, we aim to fill this knowledge gap by exploring the connection between α-HB and sepsis using multi-database data mining, machine learning, and unsupervised clustering analyses. By integrating data from multiple bioinformatics resources, including SEA, SuperPred, and SwissTargetPrediction, we identified a set of α-HB-related targets. We then cross-referenced these targets with sepsis-related gene expression profiles derived from the GEO database, revealing a set of common targets potentially implicated in sepsis progression. These targets were significantly enriched in immune response, hypoxia, and cancer-related pathways, suggesting α-HB may influence sepsis through these biological processes. Through functional enrichment analyses and protein-protein interaction (PPI) network construction, we sought to uncover the underlying biological processes and pathways influenced by α-HB in the context of sepsis. Furthermore, we applied machine learning algorithms to identify biomarkers from these targets, which demonstrated high predictive accuracy for sepsis risk in our nomogram model and may serve as potential diagnostic indicators. Figure 1 presents the flow chart of the study. By combining computational approaches with independent dataset validation, our results offer novel insights into the molecular mechanisms of α-HB in sepsis, specifically demonstrating that: (1) α-HB exposure may significantly impact disease progression through immune/inflammatory pathway modulation, as evidenced by our unsupervised clustering revealing two distinct sepsis subtypes with differential survival outcomes; (2) Molecular docking confirms α-HB’s potential interactions with key targets (APEX1, CTSD, SLC40A1, PIK3CB), providing mechanistic explanations for its biological effects.

In summary, this study aims to identify novel therapeutic targets and biomarkers that may improve the clinical management of sepsis, as well as to contribute to the growing understanding of how microbiota-derived metabolites influence host-pathogen interactions in systemic inflammatory diseases.

## Methods

### Data collection and preparation

The gene expression datasets used in this study were obtained from the Gene Expression Omnibus (GEO) database. The primary dataset, GSE26440, comprises 32 healthy control samples and 98 sepsis samples. The datasets GSE13904 (18 healthy controls and 158 sepsis samples), GSE26378 (21 healthy controls and 82 sepsis samples), GSE9692 (15 healthy controls and 30 sepsis samples), GSE8121 (15 healthy controls and 60 sepsis samples), and GSE4607 (15 healthy controls and 69 sepsis samples) were utilized for validation purposes (Supplementary Table S1). Raw data from GEO database were processed using the R programming language. The raw data from all datasets were preprocessed and normalized to ensure consistency and comparability. We used the affy package for processing Affymetrix microarray data, which included background correction, normalization, and summarization. Specifically, the Robust Multichip Average (RMA) method was employed for normalization, which corrects for background noise and normalizes the expression values across samples. Furthermore, Combat function from the sva package was used to correct for batch effects that may arise from differences in experimental conditions among the datasets.

### Identification of α-HB-related targets

To identify α-HB-related targets, we utilized three well-established databases: Similarity Ensemble Approach (SEA), SuperPred, and SwissTargetPrediction. For SwissTargetPrediction, we included targets with a probability greater than 10%. This relatively permissive threshold was chosen because SwissTargetPrediction employs a 2D and 3D similarity approach with known active ligands, and the 10% cutoff has been widely used in cheminformatics studies to capture potential targets while maintaining reasonable specificity (Daina et al., 2019). For SuperPred, we included targets with a probability greater than 50%. This more stringent threshold was selected because SuperPred predictions are based on structural similarity to known drugs, and the 50% cutoff represents a balance between capturing relevant targets and reducing false positives, as recommended by the database developers (Nickel et al., 2014). For SEA, we included targets with a p-value less than 0.05. This statistical significance threshold is standard for SEA analysis, representing targets with statistically significant similarity to known bioactive compounds in the database (Wang et al., 2016). We then proceeded to integrate gene expression data related to sepsis from the GSE26440 dataset. We applied the limma package to perform differential expression analysis, identifying differentially expressed genes (DEGs) between healthy controls and sepsis samples. Genes with a false discovery rate (FDR) < 0.05 were considered statistically significant (Gu et al., 2024). The intersection of these DEGs with the 95 α-HB-associated targets led to the identification of 42 common targets, which were considered potential candidates for further investigation in sepsis progression.

### Heatmap and protein-protein interaction (PPI) network analysis

To visualize the expression patterns of the 42 α-HB targets, we performed a heatmap analysis using the pheatmap package in R. The heatmap displayed distinct expression profiles between the normal and sepsis groups. Additionally, we constructed a PPI network for these targets using the STRING database and visualized using Cytoscape 3.9.1. This network provided insights into the potential biological interactions and pathways involved in sepsis progression.

### Functional enrichment analysis

Gene Ontology (GO) and Kyoto Encyclopedia of Genes and Genomes (KEGG) pathway enrichment analyses were conducted to gain insights into the biological functions and pathways associated with the 42 α-HB-related sepsis targets. The GO enrichment analysis was performed to classify the targets into biological processes, molecular functions, and cellular components, while the KEGG pathway analysis focused on identifying significant pathways related to immune responses, cancer, and sepsis progression. These analyses were performed using the clusterProfiler R package with a threshold of adjusted p-value <0.05.

### Biomarker identification using machine learning methods

To identify potential biomarkers for sepsis from the α-HB toxicity targets, we employed three machine learning algorithms with following parameters: (1) LASSO regression (via the glmnet package, version 4.1.7): The regularization parameter lambda (λ) was optimized using 10-fold cross-validation on the training set, exploring values from 0.001 to 1.0. The “one-standard-error rule” was applied to select the final λ to enhance generalizability. The model used α = 1 for L1 penalty and standardized predictors. (2) Random Forest (RF) (via the randomForest package, version 4.7.1.1): Hyperparameters were tuned via 5-fold cross-validation with grid search. Out-of-bag error was monitored to prevent overfitting. The final configuration used 500 trees, mtry set to the square root of feature count, and node size of 1, with gene importance ranked by mean decrease in accuracy. (3) Support Vector Machine (SVM) (via the e1071 package, version 1.7.13): Hyperparameters were optimized using nested cross-validation with grid search over cost (0.1, 1, 10, 100) and gamma (0.001, 0.01, 0.1, 1) parameters. The final model used a radial basis kernel with cost (C) = 2 and gamma (γ) = 0.015625. Model performance was evaluated using multiple metrics: AUC, sensitivity, and specificity, precision. Overlapping biomarkers were visualized via the VennDiagram package.

### Nomogram construction and validation

A nomogram was constructed based on the expression profiles of the four identified biomarker genes (APEX1, CTSD, SLC40A1, and PIK3CB) to predict the progression of sepsis in patients. Violin plots were used to visualize the differential expression of these genes in sepsis patients compared to healthy controls in the three datasets. The predictive accuracy of the nomogram was assessed using receiver operating characteristic (ROC) curve analysis, with the area under the curve (AUC) calculated to evaluate the model’s performance. For ROC curve analysis, an optimal threshold was determined using the Youden index to maximize the sum of sensitivity and specificity. The calibration curve was used to assess the agreement between predicted and actual outcomes, while decision curve analysis (DCA) was performed to assess the clinical utility of the nomogram across different risk thresholds.

### Clustering and enrichment analysis of sepsis subtypes

To explore the impact of α-HB on sepsis progression further, we utilized the 42 α-HB-related targets to cluster sepsis patients into distinct subtypes using the ConsensusClusterPlus package. Principal Component Analysis (PCA) was conducted using the prcomp function to visualize the separation between subtypes. Differentially expressed genes (DEGs) between the two subtypes were subjected to GO and KEGG enrichment analyses to identify distinct biological processes and pathways involved in the progression of sepsis in each subtype.

### Statistical analysis of clinical outcomes

Binary clinical outcomes (e.g., survivor vs. non-survivor) were compared between sepsis subtypes using Chi-square tests to assess significant differences in mortality rates. Results were visualized as stacked bar chart generated with the ggplot2 (version 3.4.4) and ggalluvial (version 0.12.3) packages, illustrating the distribution of outcomes across subtypes.

### Pathway activity evaluation

The Gene Set Variation Analysis (GSVA) was performed using the GSVA package to evaluate pathway activities between the identified α-HB-related sepsis subtypes. Heatmaps were generated to visualize distinct pathway activity profiles. Pathway significance levels were determined using a false discovery rate (FDR) threshold of <0.05 to identify significantly altered pathways.

### Immune cell infiltration analysis

The single-sample Gene Set Enrichment Analysis (ssGSEA) was conducted using the GSEABase and GSVA packages to evaluate immune cell infiltration differences between the two α-HB-related sepsis subtypes. This approach allowed for the quantification of immune cell infiltration levels in each sample. A heatmap was generated to illustrate the differences in immune cell infiltration between the two subtypes, and box plots were used to visualize the infiltration patterns of various immune cell types.

### Molecular docking of α-HB with key targets

Molecular docking studies were conducted to explore the interactions between α-HB and the four key sepsis biomarkers (APEX1, CTSD, SLC40A1, and PIK3CB). The docking simulations were performed using AutoDock Vina (version 1.5.7). Initially, the 3D structures of the target proteins were obtained from the Protein Data Bank (PDB) (https://www.rcsb.org/), and any water molecules, heteroatoms, or bound ligands were removed using PyMOL (version 2.5.2) to prepare the proteins for docking. The structure of α-HB was downloaded from the PubChem database (https://pubchem.ncbi.nlm.nih.gov/), and its 3D coordinates were converted to PDBQT format using AutoDockTools. The ligand was then energy-minimized to ensure stability. A 3D grid box was generated around each protein’s active site in AutoDockTools, with careful adjustment of the grid dimensions to encompass the binding site. Docking evaluation was based on the binding affinity scores, with lower energy values indicating stronger binding interactions. The top binding poses were selected based on their energy values, and the results were visualized in PyMOL.

## Results

### Identification and analysis of α-HB-related targets in sepsis

**FIGURE 2:**
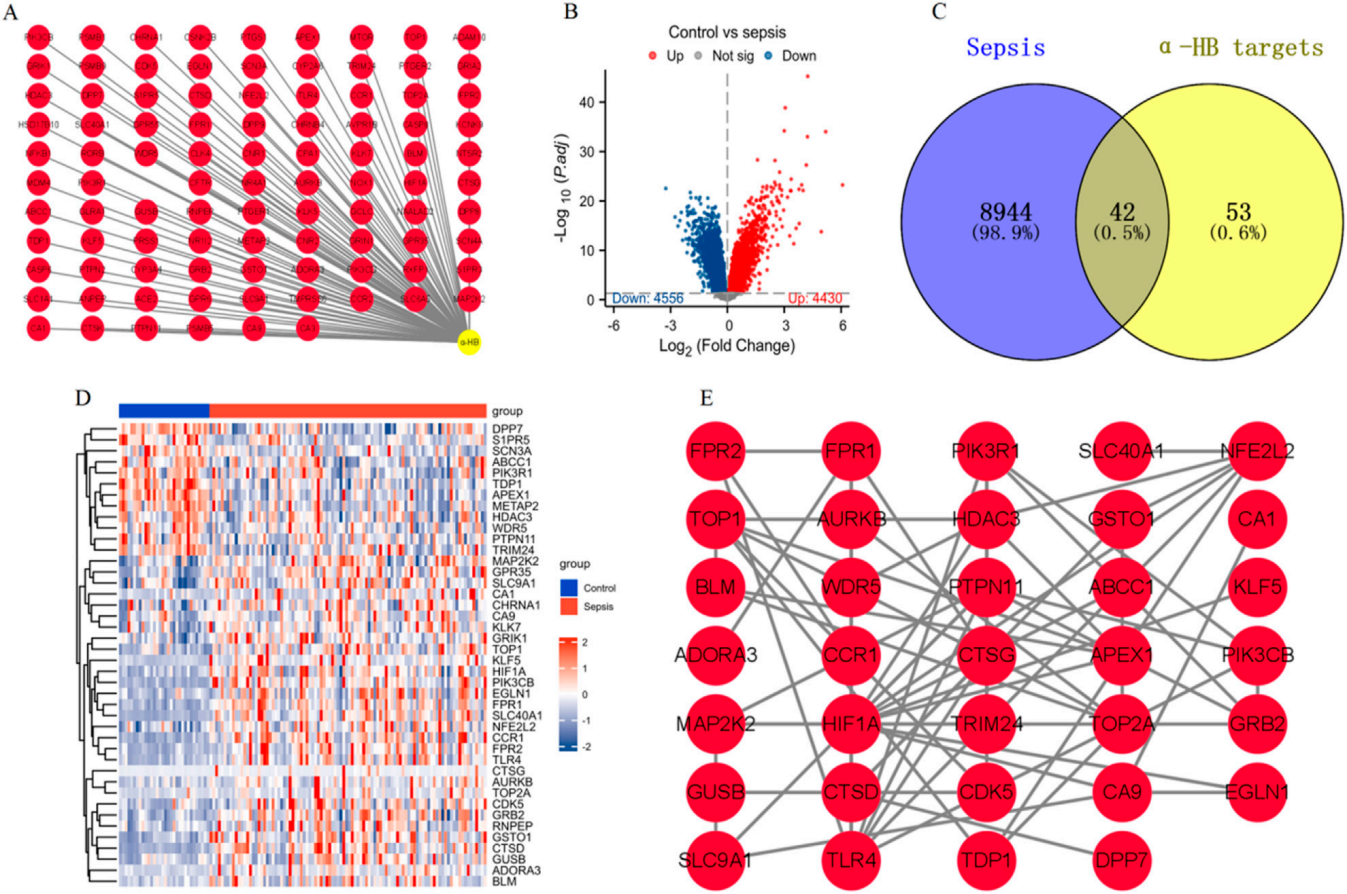
Identification and analysis of α-HB-related targets in the context of sepsis. **(A)** α-HB related targets were collected from the SEA, SuperPred, and SwissTargetPrediction databases. **(B)** Differential expression analysis of sepsis-related dataset GSE26440 from the GEO database. **(C)** Intersection of sepsis-associated differentially expressed genes and α-HB related targets revealed 42 common targets potentially involved in sepsis progression. **(D)** Heatmap analysis displaying the expression profiles of the 42 intersecting α-HB targets. **(E)** PPI network of the 42 common targets.

In this study, we identified a total of 95 α-HB-related targets through comprehensive data mining from three databases: SEA, SuperPred, and SwissTargetPrediction (Figure 2A). Subsequently, we analyzed the gene expression profiles associated with sepsis by downloading the dataset GSE26440 from the GEO database. We identified 8,986 sepsis-related targets, highlighting significant alterations in gene expression between the normal and disease groups (Figure 2B). To further elucidate the relationship between α-HB and sepsis progression, we intersected the identified sepsis-related targets with the α-HB related targets. This analysis revealed 42 common targets that are potentially implicated in the progression of sepsis (Figure 2C). To visualize the expression patterns of these 42 α-HB targets, we performed a heatmap analysis, which demonstrated distinct expression profiles between the normal and sepsis groups (Figure 2D). Additionally, we constructed a protein-protein interaction (PPI) network for these targets, providing insights into the potential biological interactions and pathways involved in sepsis progression (Figure 2E). These findings underscore the critical role of α-HB in modulating the molecular landscape of sepsis, suggesting that these identified targets may serve as potential biomarkers or therapeutic targets for managing sepsis.

### The functional enrichment analysis of the 42 α-HB-related targets

**FIGURE 3:**
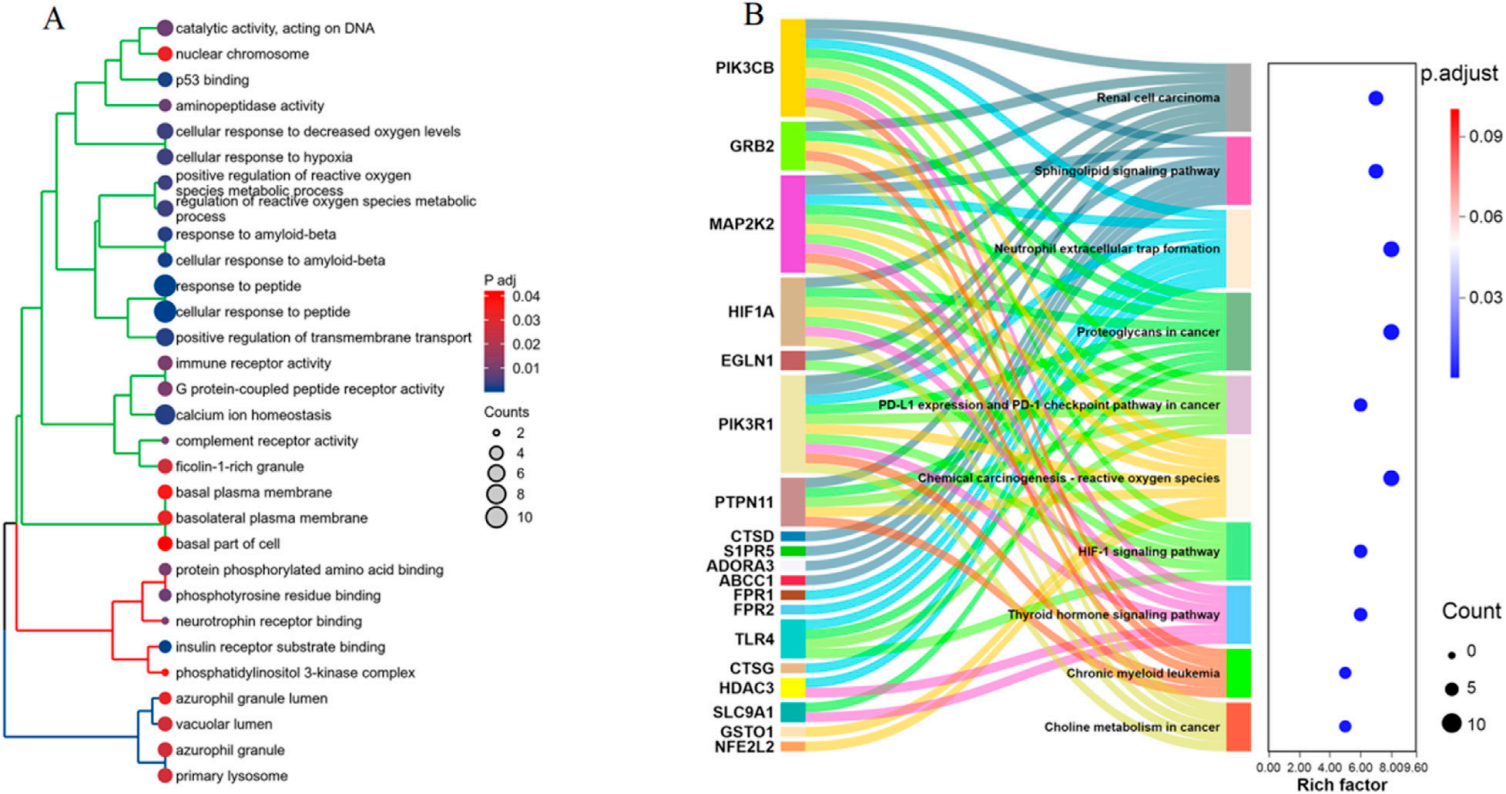
Enrichment analysis of the 42 α-HB targets. **(A)** GO enrichment analysis of the 42 α-HB targets, showing significant enrichment in various biological processes, molecular functions, and cellular components. **(B)** KEGG pathway enrichment analysis of the 42 α-HB targets. The dot plot on the right illustrates the significance (adjusted p-value) and the number of genes involved in each pathway.

GO enrichment analysis highlighted several enriched biological processes, molecular functions, and cellular components. Notably, the targets were significantly associated with processes such as “cellular response to hypoxia,” “regulation of reactive oxygen species metabolic process,” and “immune receptor activity.” Molecular functions included “p53 binding,” “phosphotyrosine residue binding,” and “G protein-coupled peptide receptor activity.” Key cellular components identified were “nuclear chromosome,” “basolateral plasma membrane,” and “primary lysosome” (Figure 3A). KEGG pathway analysis revealed significant enrichment in several pathways linked to cancer and immune response. Noteworthy pathways included “PD-L1 expression and PD-1 checkpoint pathway in cancer,” “HIF-1 signaling pathway,” and “Sphingolipid signaling pathway.” Additionally, pathways such as “Chemical carcinogenesis - reactive oxygen species,” (Supplementary Figure S1) “Neutrophil extracellular trap formation,” (Supplementary Figure S2) and “Renal cell carcinoma” were also significantly associated with the identified targets (Figure 3B). These enrichment analyses provide comprehensive insights into the potential mechanisms by which α-HB may influence sepsis progression, highlighting critical biological processes and pathways that warrant further investigation.

### Identification of biomarker genes for sepsis from the α-HB-related targets using machine learning algorithms

**FIGURE 4:**
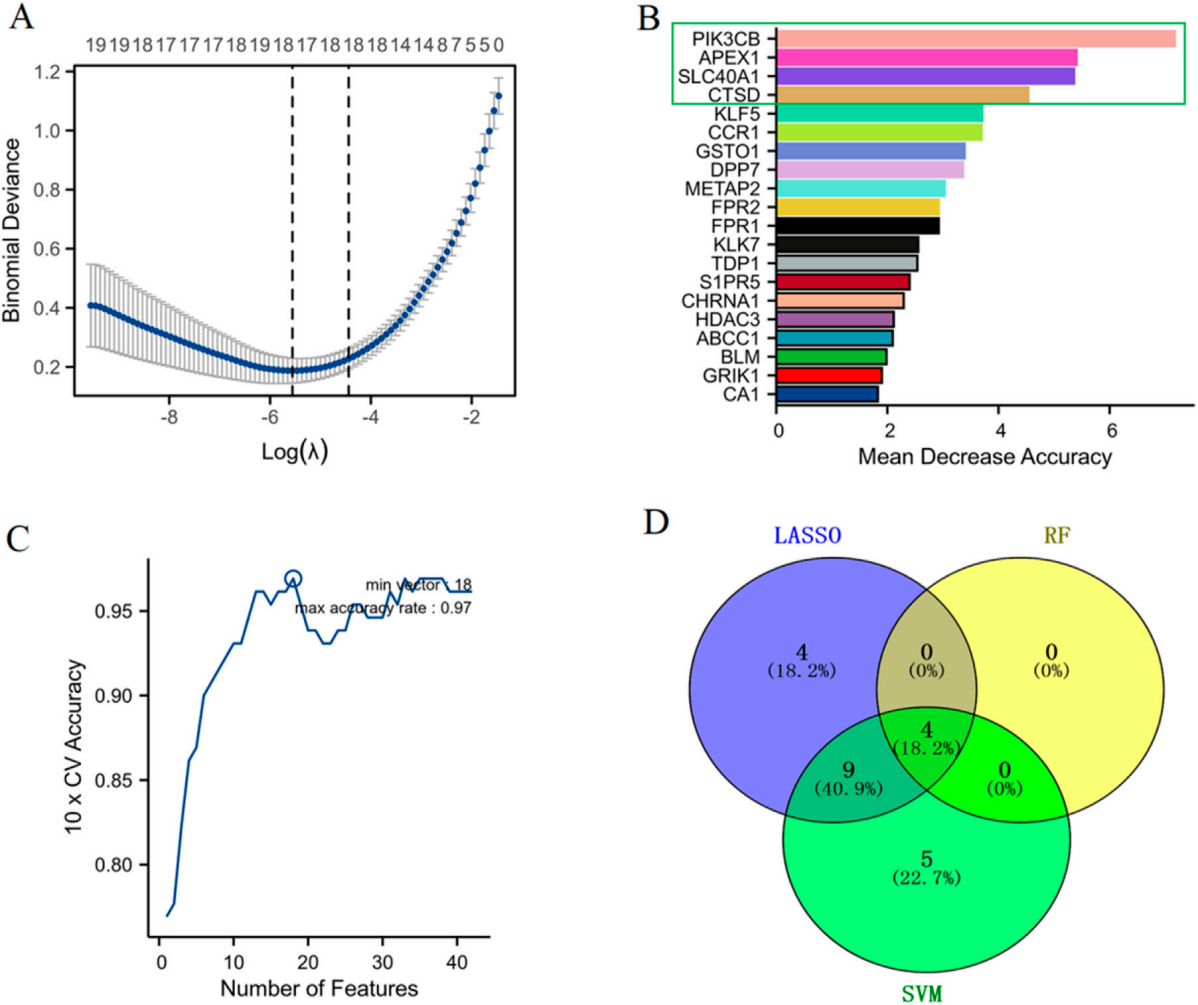
Identification of biomarker genes for sepsis. **(A)** LASSO regression analysis determined the optimal lambda (λ) value through cross-validation, allowing the selection of the most relevant features (genes) by minimizing binomial deviance. **(B)** RF analysis ranked gene importance based on the mean decrease in accuracy. **(C)** SVM performance was assessed via 10-fold cross-validation, with accuracy plotted against the number of features used, showing that optimal model performance was achieved with a certain subset of features. **(D)** The Venn diagram shows the distribution of selected biomarker genes across the three machine learning algorithms.

The optimal lambda (λ) value was determined through cross-validation, significantly narrowing down the number of features (genes) (Figure 4A). The RF algorithm measured the mean decrease in accuracy for each gene, providing an importance ranking. Four genes (APEX1, CTSD, SLC40A1, and PIK3CB) were identified as the most crucial markers based on their significant impact on model accuracy (Figure 4B). The performance of the SVM algorithm was evaluated by 10-fold cross-validation, where the accuracy increased with the number of features and finally reached a high point where 18 feature genes were identified (Figure 4C). A Venn diagram illustrates the commonality and differences in biomarkers identified by each algorithm. Notably, APEX1, CTSD, SLC40A1, and PIK3CB were consistently selected by all three algorithms, underscoring their potential as robust marker genes for sepsis diagnosis and progression (Figure 4D). These results demonstrate the effectiveness of combining multiple machine learning methods to identify robust biomarkers from α-HB targets, providing valuable insights for the early detection and treatment of sepsis.

### Construction and validation of a nomogram

**FIGURE 5:**
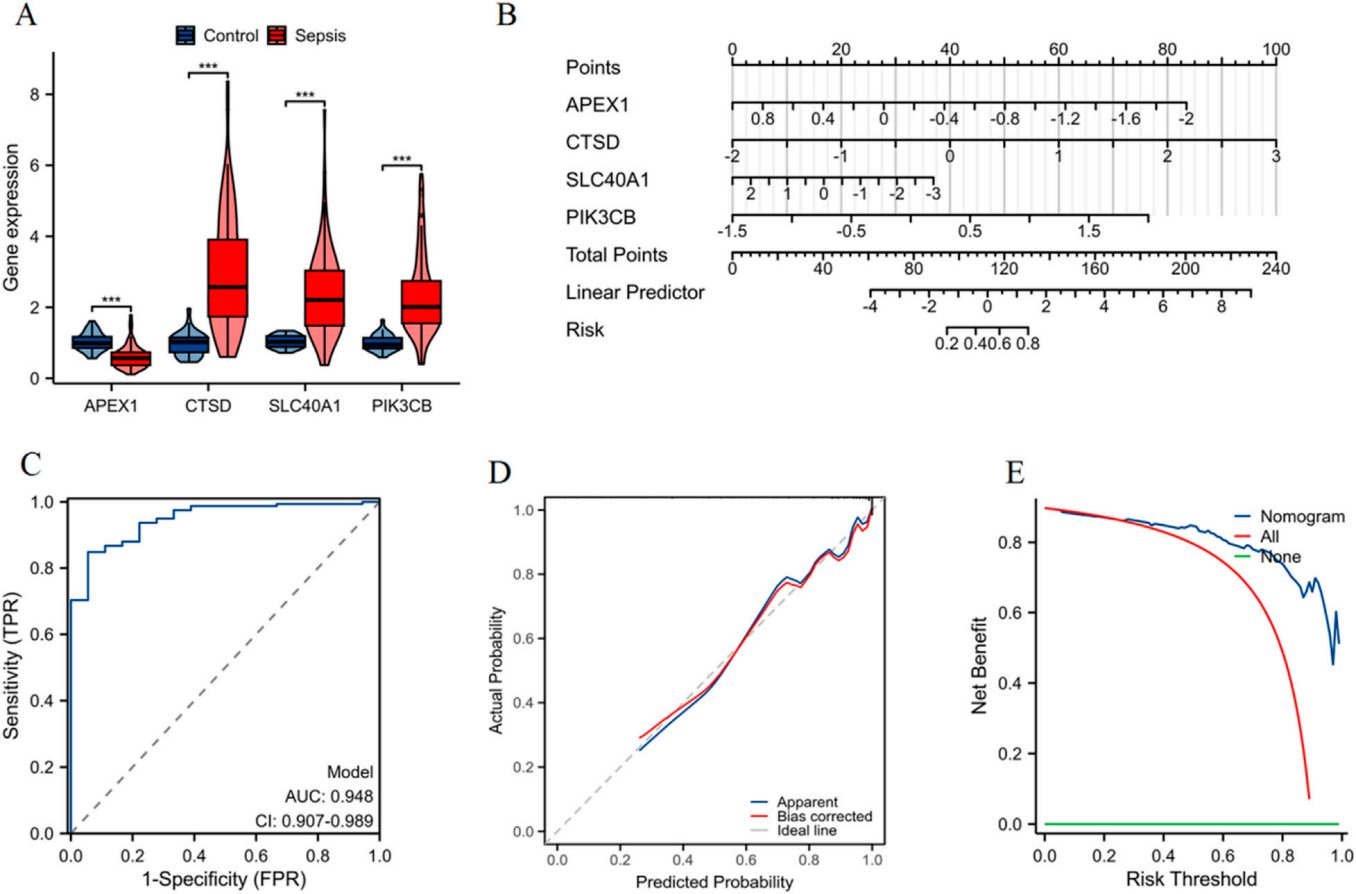
Construction and validation of a nomogram for sepsis patients. **(A)** Violin plots showing the differential expression of APEX1, CTSD, SLC40A1, and PIK3CB between control and sepsis groups (***p < 0.001). **(B)** Nomogram constructed to predict the risk of sepsis, with each gene contributing to an overall risk score. **(C)** ROC curve indicating high predictive accuracy of the nomogram. **(D)** Calibration curve demonstrating a strong correlation between predicted probability and actual probability, confirming the model’s predictive accuracy. **(E)** DCA illustrating the net benefit of using the nomogram across different risk thresholds, compared to treating all patients or none.

As shown in Figure 5A, violin plots depict the differential expression of CTSD, SLC40A1, PIK3CB (upregulated), and APEX1 (downregulated) in sepsis patients compared to the control group (***p < 0.001). In addition, we validated the expression of these four diagnostic genes in independent datasets GSE13904, GSE26378, GSE9692, GSE8121, and GSE4607 (Supplementary Figure S3). A nomogram was constructed based on the expression profiles of APEX1, CTSD, SLC40A1, and PIK3CB, allowing for individualized prediction of sepsis risk (Figure 5B). ROC curve of the model indicates high predictive accuracy with an AUC of 0.948 (95% CI: 0.907–0.989) (Figure 5C). The calibration curve shows good agreement between predicted probabilities and actual outcomes, confirming the model’s predictive validity (Figure 5D). DCA demonstrates that the nomogram offers a higher net benefit across different risk thresholds compared to treating all patients or none, indicating its clinical utility (Figure 5E). These findings validate the use of the nomogram based on these four genes for accurately assessing sepsis risk in patients.

### Validation of diagnostic performance of four genes across five independent datasets

**FIGURE 6:**
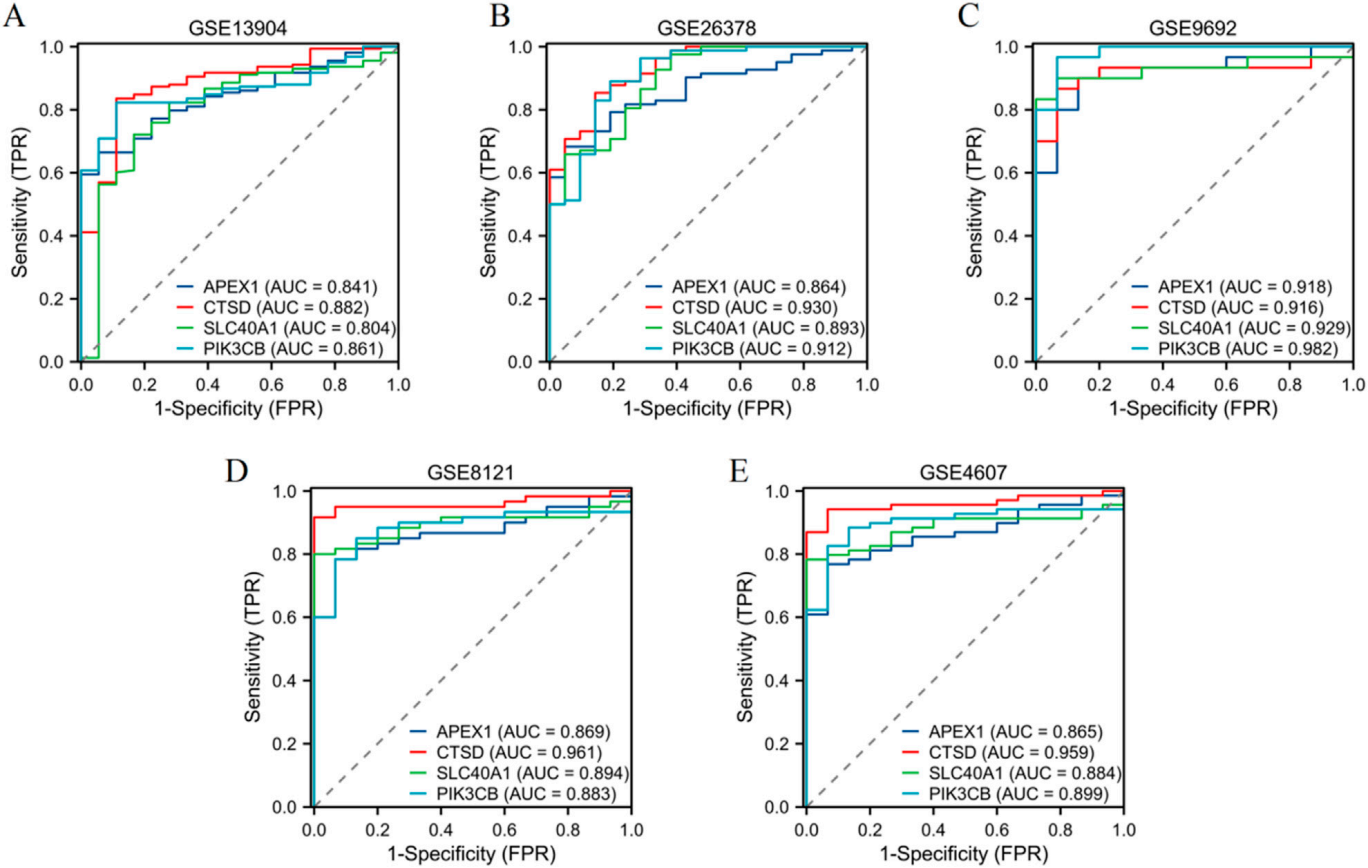
Validation of diagnostic performance of four genes. ROC curves illustrating the diagnostic performance of four genes (APEX1, CTSD, SLC40A1, PIK3CB) in five independent datasets: **(A)** GSE13904, **(B)** GSE26378, **(C)** GSE9692, **(D)** GSE8121, and **(E)** GSE4607.

In the GSE13904 dataset (Figure 6A), the AUC values were as follows: APEX1 (AUC = 0.841), CTSD (AUC = 0.882), SLC40A1 (AUC = 0.804), and PIK3CB (AUC = 0.861). For the GSE26378 dataset (Figure 6B), APEX1 achieved an AUC of 0.864, CTSD 0.930, SLC40A1 0.893, and PIK3CB 0.912. The GSE9692 dataset (Figure 6C) showed higher AUC values: APEX1 (AUC = 0.918), CTSD (AUC = 0.916), SLC40A1 (AUC = 0.929), and PIK3CB (AUC = 0.982). In the GSE8121 dataset (Figure 6D), the AUC for APEX1 was 0.869, CTSD 0.961, SLC40A1 0.894, and PIK3CB 0.883. Finally, in the GSE4607 dataset (Figure 6E), APEX1 showed an AUC of 0.865, CTSD 0.959, SLC40A1 0.884, and PIK3CB 0.899. These results demonstrate the robust diagnostic capabilities of the four genes across multiple datasets, with consistently high AUC values.

### Clustering and enrichment analysis of α-HB-related sepsis subtypes

**FIGURE 7:**
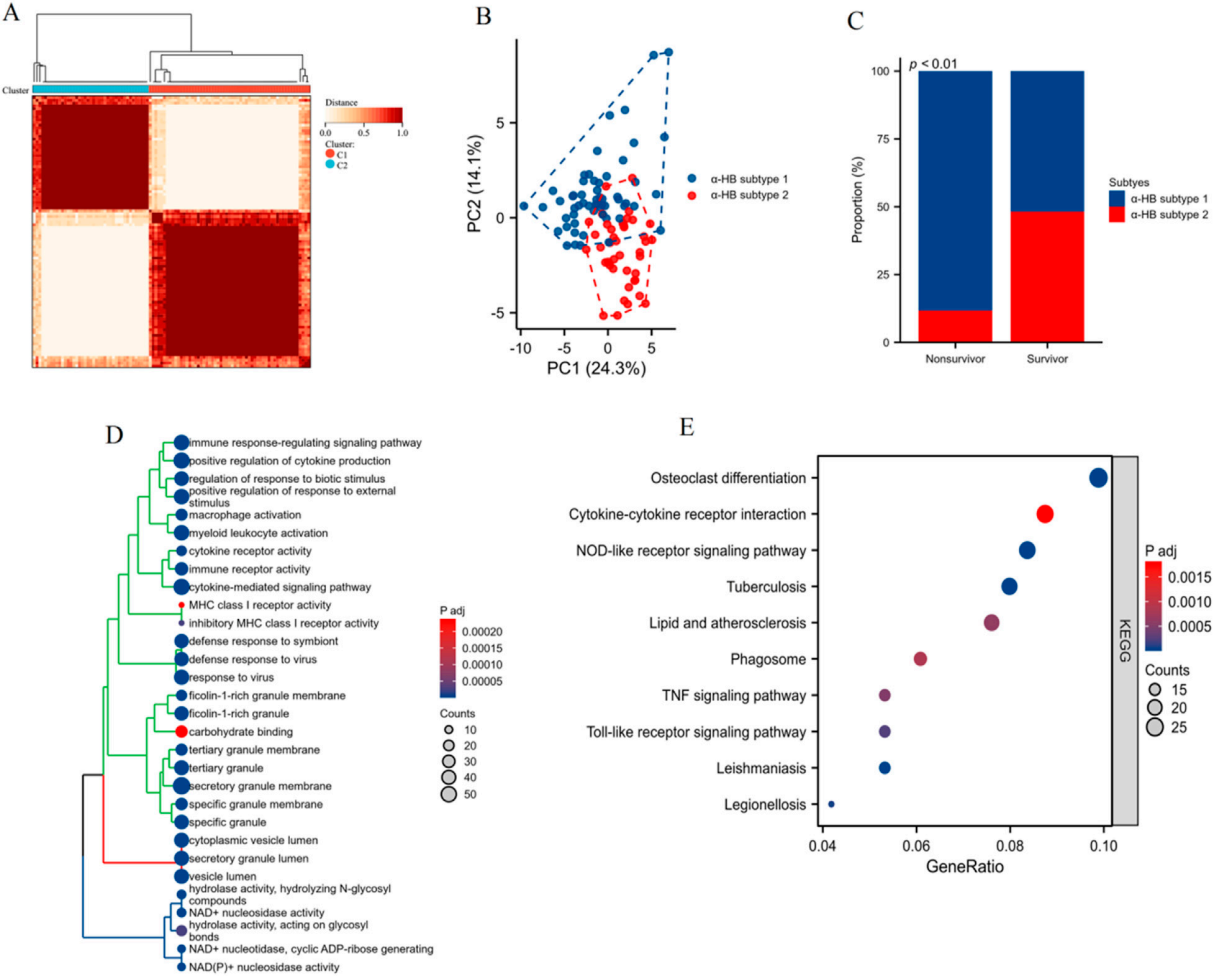
Clustering and enrichment analysis of sepsis patients based on the α-HB toxicity targets. **(A)** Heatmap showing the clustering of sepsis patients into two distinct α-HB-related disease subtypes (HB subtype 1 and HB subtype 2) based on the expression profiles of the 42 α-HB-related targets. **(B)** Principal Component Analysis (PCA) plot confirming the separation between HB subtype 1 and HB subtype 2. **(C)** Bar graph illustrating the proportion of non-survivors and survivors in each α-HB-related sepsis subtype. **(D)** GO enrichment analysis of differentially expressed genes between the two α-HB-related subtypes. **(E)** KEGG pathway enrichment analysis illustrating significantly enriched pathways between the two subtypes.

As shown in Figure 7A, clustering analysis based on the expression profiles of the 42 α-HB targets identified two distinct α-HB-related disease subtypes, referred to as HB subtype 1 and HB subtype 2. Principal Component Analysis (PCA) confirmed clear separation between HB subtype 1 and HB subtype 2, accounting for 24.3% and 14.1% of the total variance, respectively (Figure 7B). Figure 7C presents the survival status analysis, highlighting a significant difference (p < 0.01) in survival outcomes between the two α-HB-related sepsis subtypes. α-HB Subtype 1 is predominantly associated with non-survivors, whereas α-HB Subtype 2 is more frequent among survivors. Differentially expressed genes between the two α-HB-related subtypes were subjected to GO enrichment analysis. The analysis showed enrichment in biological processes such as immune response-regulating signaling pathway, cytokine production regulation, macrophage activation, and cytokine receptor activity (Figure 7D). KEGG pathway enrichment analysis of the DEGs between the two subtypes highlighted several significantly enriched pathways, including Osteoclast differentiation, Cytokine-cytokine receptor interaction, NOD-like receptor signaling pathway, TNF signaling pathway, and Toll-like receptor signaling pathway (Figure 7E). These analyses reveal two distinct molecular subtypes of sepsis influenced by α-HB exposure, each characterized by different gene expression profiles and enriched pathways, providing insights into potential mechanisms driving sepsis progression and informing targeted therapeutic strategies.

### Pathway activity evaluation between α-HB-related sepsis subtypes employing GSVA

**FIGURE 8:**
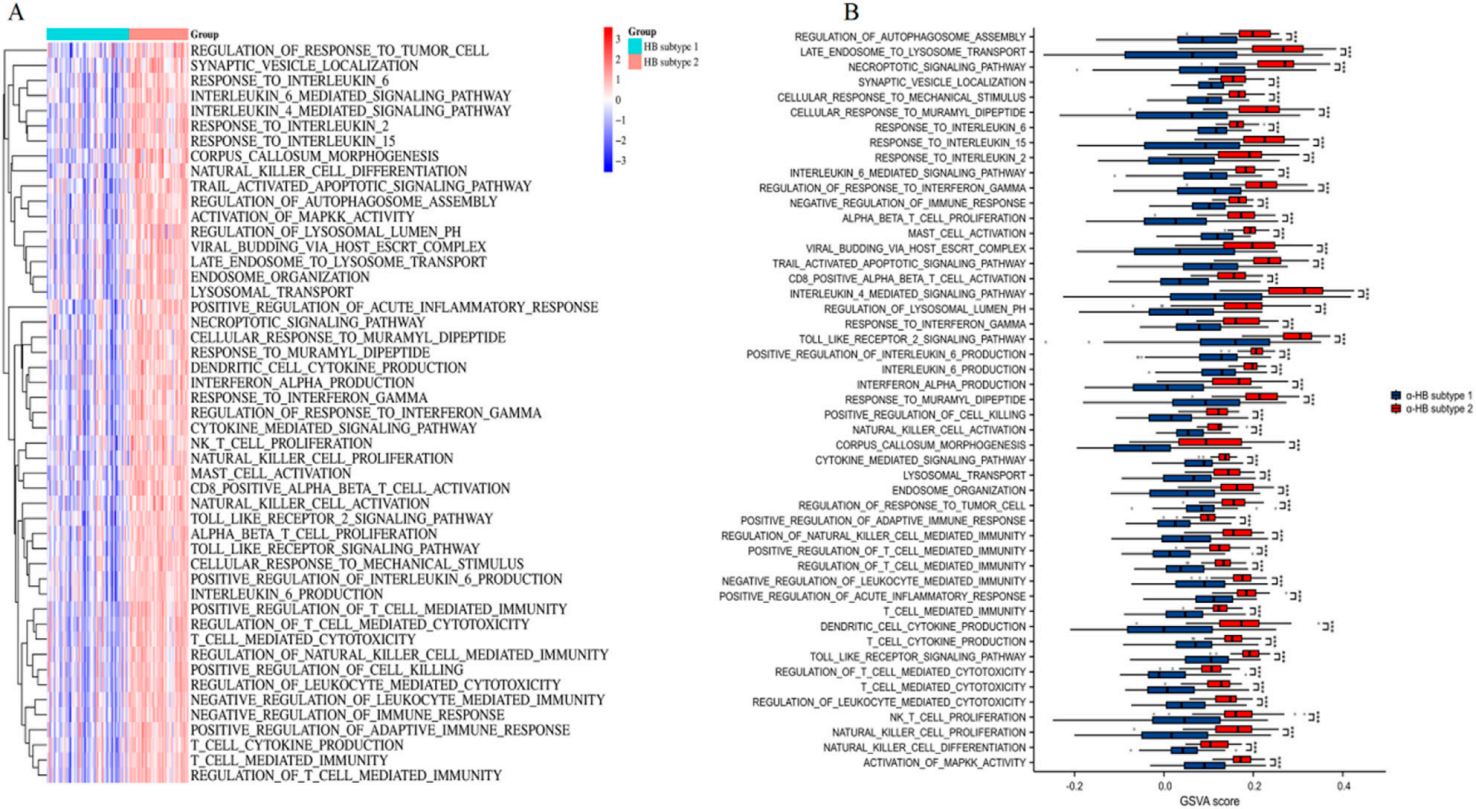
Pathway activity assessment between α-HB-related sepsis subtypes using GSVA. **(A)** Heatmap of GSVA scores illustrating distinct pathway activities between α-HB subtype 1 and α-HB subtype 2. **(B)** Box plots comparing GSVA scores for various pathways between the two α-HB-related subtypes. ***p < 0.001.

As shown in Figure 8A, the heatmap of GSVA scores shows distinct pathway activity profiles between α-HB subtype 1 and α-HB subtype 2. Notably, α-HB subtype 2 exhibited significant enrichment in various immune and inflammatory pathways, including regulation of response to tumor cells, synaptic vesicle localization, and multiple interleukin-mediated signaling pathways (such as IL-6, IL-4, IL-2, and IL-15). Box plots further illustrate the significant differences in GSVA scores for various pathways between the two subtypes (Figure 8B). Immune and inflammatory pathways were prominently enriched in α-HB subtype 2, including: regulation of autophagosome assembly, response to interleukins (e.g., IL-6, IL-2, IL-15), regulation of response to interferon gamma and cytokine-mediated signaling pathways, natural killer (NK) cell proliferation and activation, and toll-like receptor signaling pathway. Overall, the GSVA analysis underscores significant pathway-level differences between α-HB subtype 1 and α-HB subtype 2. The pronounced enrichment of immune and inflammatory pathways in α-HB subtype 2 suggests distinct molecular mechanisms underlying sepsis progression in this subtype. These findings provide valuable insights for potential therapeutic targets and interventions tailored to each α-HB-related sepsis subtype.

### Analysis of immune cell infiltration between α-HB-related sepsis subtypes via ssGSEA

**FIGURE 9:**
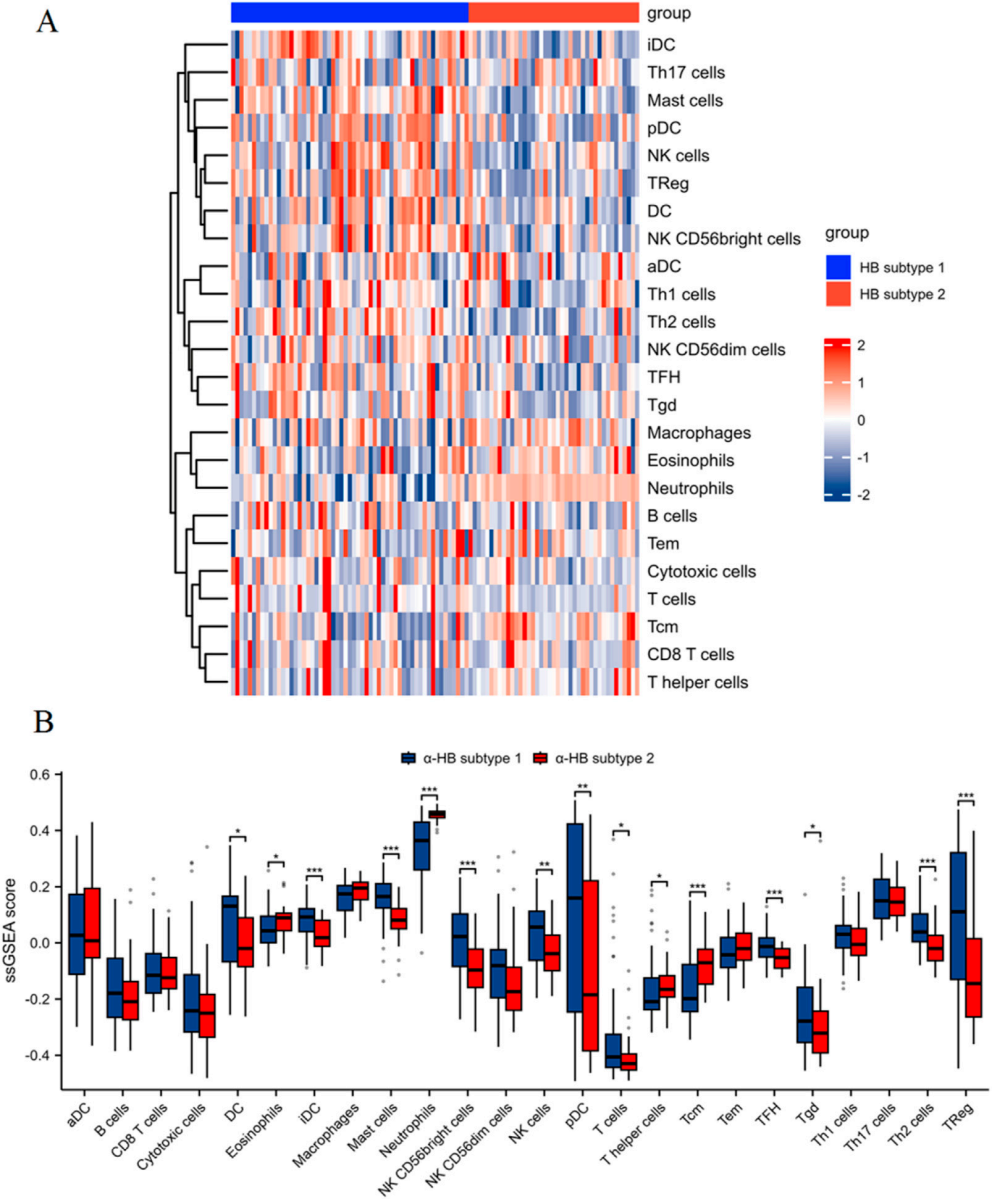
Immune cell infiltration analysis between α-HB-related sepsis subtypes using ssGSEA. **(A)** Heatmap of ssGSEA scores illustrating different levels of immune cell infiltration between α-HB subtype 1 and α-HB subtype 2. **(B)** Box plots quantifying the ssGSEA scores for various immune cell populations between the two subtypes. *p < 0.05, **p < 0.01, ***p < 0.001.

The heatmap (Figure 9A) of ssGSEA scores highlights distinct immune cell infiltration patterns, with differences observed across various immune cell types. Specifically, α-HB subtype 1 exhibited significantly higher infiltration levels of DC, iDC, pDC, mast cells, Tregs, NK cells (particularly the CD56bright subset), and TFH, all of which play crucial roles in antigen presentation, immune tolerance, innate immune response, and inflammation. In contrast, α-HB subtype 2 showed higher infiltration levels of neutrophils and eosinophils, which are key players in acute inflammation, phagocytosis, and allergic responses, respectively. The box plots (Figure 9B) quantitatively confirm these differences, illustrating the distinct immunological landscapes between the two subtypes. These findings suggest that the differential immune cell infiltration likely contributes to the distinct progression and treatment response profiles observed in the α-HB-related sepsis subtypes, indicating potential avenues for targeted immunotherapeutic strategies.

### Molecular docking analysis of α-HB with key targets

**FIGURE 10:**
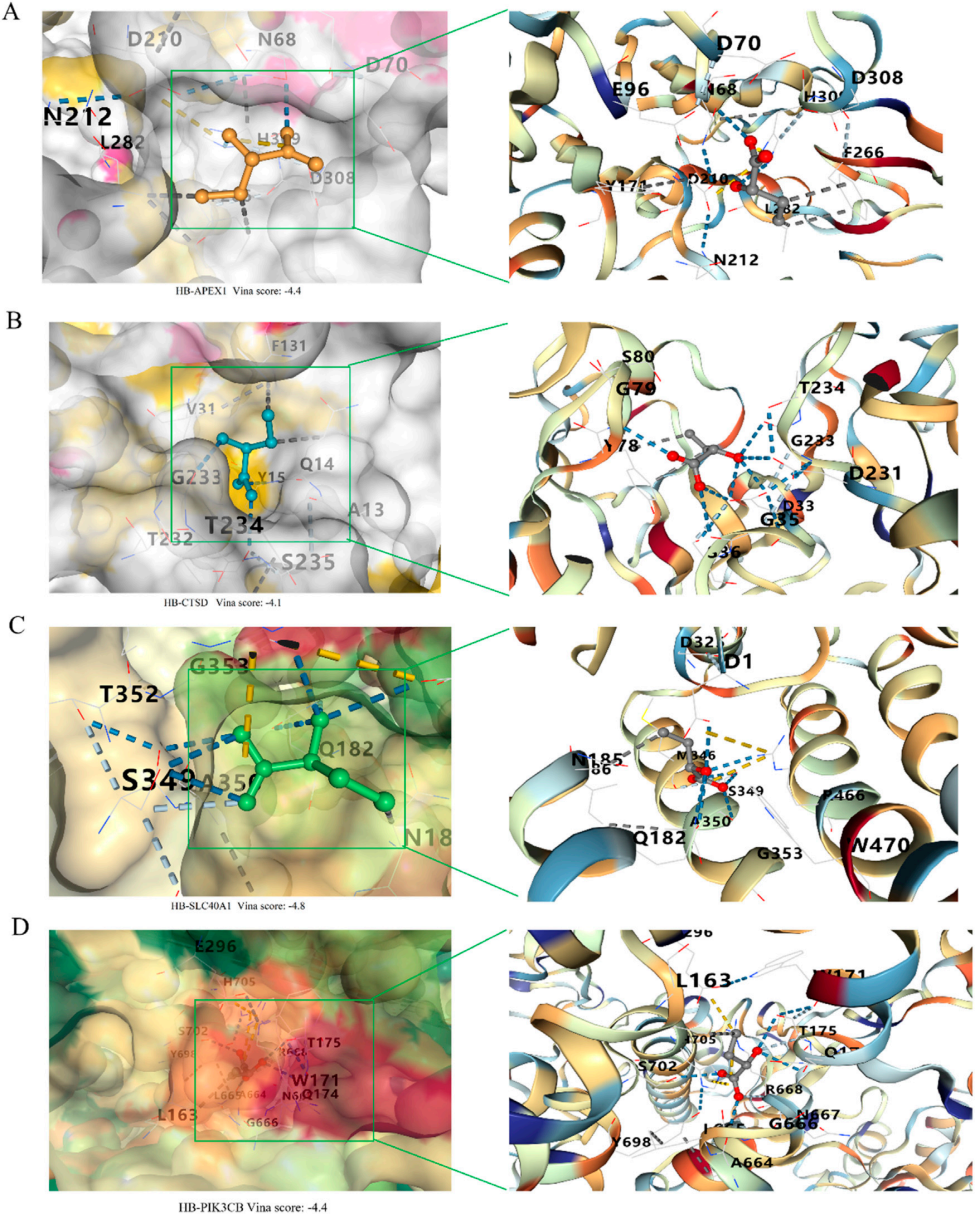
Molecular docking analysis of α-HB with key targets. **(A)** Docking of α-HB with APEX1. **(B)** Docking of α-HB with CTSD. **(C)** Docking of α-HB with SLC40A1. **(D)** Docking of α-HB with PIK3CB.

The docking results, including Vina scores (representing binding affinity), key interacting residues, and docking grid dimensions, are summarized in Supplementary Table S2. As shown in Figure 10A, the molecular docking of α-HB with APEX1 revealed a binding affinity with a Vina score of −4.4. Key interactions included hydrogen bonds and hydrophobic interactions with residues D308, H309, F266, L282 and N212. Docking analysis of α-HB with CTSD showed a binding affinity with a Vina score of −4.1. The docking model identified potential interactions with residues G35, D33, G233, D231 and T234 (Figure 10B). The interaction of α-HB with SLC40A1 was identified with a Vina score of −4.8, exhibiting hydrogen bonding and hydrophobic interactions with residues R466, W470, G353, A350, Q182, and N185 (Figure 10C). The docking study of α-HB with PIK3CB indicated a binding affinity with a Vina score of −4.4. The interactions involved key residues Y698, A664, G666, N667, R668 and Q174 (Figure 10D). These molecular docking results suggest that α-HB can potentially interact with key targets involved in sepsis progression. The identified interactions provide insights into the molecular mechanisms by which α-HB might influence the function of these targets, contributing to the overall understanding of its role in sepsis pathophysiology.

## Discussion

The results of this study provide novel insights into the role of α-HB, a short-chain fatty acid derived from gut microbiota, in modulating sepsis progression through its interaction with key molecular targets and pathways. Our comprehensive approach, integrating bioinformatics, machine learning algorithms and functional enrichment analyses, has identified α-HB’s targets, potential biomarkers for sepsis, and disease subtypes influenced by α-HB exposure. However, it is important to acknowledge the primarily correlational nature of our findings, and that several potential confounders and indirect associations merit consideration when interpreting these results. While these findings provide valuable insights into how α-HB relates to sepsis pathophysiology, a condition characterized by dysregulated immune responses and systemic inflammation, key limitations warrant emphasis. Our analysis identifies associations between α-HB targets and sepsis pathways, yet potential confounding factors (e.g., patient demographics, underlying comorbidities, medication use, and sepsis etiological heterogeneity) may influence both α-HB metabolism and clinical outcomes. Furthermore, the observed relationships may reflect indirect effects mediated through complex host-microbiome metabolic networks rather than direct causal mechanisms.

Functional enrichment analysis highlighted several key biological processes, molecular functions, and cellular components that are associated with α-HB exposure. These include cellular response to hypoxia, regulation of reactive oxygen species (ROS) metabolic process, and immune receptor activity, all of which are critical in sepsis pathophysiology. Our findings align with previous studies indicating metabolic alterations, oxidative stress, and immune dysfunction as key players in sepsis progression (Ingels et al., 2018; Bavunoglu et al., 2016). The association of α-HB with cellular response to hypoxia suggests that α-HB could modulate tissue hypoxia, a common feature in sepsis due to impaired perfusion and oxygen delivery (Bar-Or et al., 2015). The mechanistic basis for this association could involve α-HB’s interference with mitochondrial respiratory chain complexes or its ability to stabilize hypoxia-inducible factors through direct protein interactions or epigenetic modifications. Hypoxic stress in sepsis triggers the activation of hypoxia-inducible factors, which regulate immune cell activation and inflammation (Vanderhaeghen et al., 2020). Our findings imply that α-HB may influence this response, potentially affecting both the inflammatory cascade and the resolution of inflammation in septic tissues. This is supported by studies showing gut microbiota and its metabolites can influence hypoxic responses, underscoring the microbiome’s role in sepsis progression (Pral et al., 2021).

The identification of α-HB’s association with ROS metabolism is highly relevant to sepsis, where excessive ROS production contributes to cellular damage, organ failure, and immune suppression (Lopes-Pires et al., 2021). Modulation of ROS by α-HB might influence oxidative stress pathways and potentially protect against ROS-induced damage. This aligns with studies showing protective effects of other microbiota-derived metabolites, such as short-chain fatty acids, in mitigating oxidative stress during inflammatory conditions (Al-Harbi et al., 2018). The role of α-HB in ROS regulation presents an interesting therapeutic target for managing oxidative damage in sepsis. Moreover, the association of α-HB with immune receptor activity suggests its potential role in immune modulation. Sepsis is characterized by an initial hyperinflammatory response, followed by immune dysfunction and suppression, leading to increased susceptibility to secondary infections (Chen H. et al., 2023). α-HB’s potential to modulate immune receptor activity could influence immune cell signaling and the balance between pro-inflammatory and anti-inflammatory responses. This is consistent with findings that gut-derived metabolites, including butyrate and other short-chain fatty acids, regulate immune responses by modulating immune cell function and cytokine production (Ney et al., 2023; Wu et al., 2020).

The KEGG pathway analysis revealed significant enrichment of α-HB targets in pathways linked to cancer and immune responses, such as PD-L1 expression and PD-1 checkpoint pathway, HIF-1 signaling pathway, and Sphingolipid signaling pathway. These pathways are particularly relevant to sepsis, as immune dysregulation and tissue inflammation are central to both sepsis progression and cancer immunity (Cao et al., 2023). The observed enrichment of PD-1/PD-L1 signaling is intriguing and suggests a testable hypothesis that α-HB could potentially modulate immune tolerance and contribute to immune cell exhaustion in sepsis (Kotanides et al., 2020; Liechtenstein et al., 2012). Excessive PD-1 activation can lead to immune exhaustion, impairing host defense against infections. Several studies have implicated PD-1/PD-L1 signaling in suppressing immune function during sepsis (Chen Y. et al., 2023; Nakamori et al., 2020), though this mechanistic link requires experimental validation to establish causality between α-HB and PD-1/PD-L1 regulation. The involvement of the HIF-1 signaling pathway, central in cellular adaptation to hypoxia and inflammation (Balamurugan, 2016), supports the hypothesis that α-HB may influence immune and metabolic responses during sepsis. Hypoxia-induced activation of HIF-1 in sepsis can drive both inflammatory responses and tissue repair mechanisms (Ruan et al., 2023). The intersection between α-HB and HIF-1 signaling underscores α-HB’s potential as a modulator of immune and metabolic processes during sepsis. Additionally, the sphingolipid signaling pathway’s enrichment among α-HB toxicity targets is relevant to sepsis. Sphingolipids, like ceramides, are bioactive molecules that regulate immune cell activation, inflammation, and apoptosis (Chan et al., 2014; Patwardhan et al., 2016). In sepsis, sphingolipid metabolism regulates immune responses and influences the balance between pro-inflammatory and anti-inflammatory signaling (Hering et al., 2024).

The application of machine learning algorithms identified biomarker genes from the α-HB targets, representing a significant advancement in sepsis diagnostics. The consistent identification of APEX1, CTSD, SLC40A1, and PIK3CB as crucial markers underscores their potential clinical utiliy. APEX1 is known for its role in DNA repair and cellular stress responses (Maia de Oliveira da Silva et al., 2019). Similarly, SLC40A1 regulates iron homeostasis and is associated with inflammatory responses in critically ill patients (Wang et al., 2023). CTSD is involvement in macrophage activation (Ruiz-Blázquez et al., 2024), while PIK3CB, part of the PI3K signaling cascade, is associated with immune and inflammatory processes (Ye and Zeng, 2022). Their recurrent recognition as biomarkers underscores their significance in sepsis. These findings align with previous research using machine learning for biomarker discovery in sepsis (Komorowski et al., 2022). By integrating α-HB targets into machine learning models, our study provides a unique perspective on how gut microbiota metabolites may influence sepsis progression through their interactions with key molecular pathways.

Our clustering analysis revealed two distinct α-HB-related sepsis subtypes, each characterized by unique gene expression profiles and enriched pathways. This stratification of sepsis patients aligns with the growing recognition of sepsis as a heterogeneous syndrome. Previous studies have categorized sepsis patients based on genomic and transcriptomic data, highlighting the potential for personalized medicine in sepsis management (Lukaszewski et al., 2022). Among the significantly enriched pathways identified between the subtypes in our study, we hypothesize that the NOD-like receptor signaling pathway, TNF signaling pathway, and Toll-like receptor signaling pathway are modulated by α-HB. The NOD-like receptor signaling pathway is crucial for detecting intracellular pathogens and initiating immune responses, and its modulation by α-HB could influence the inflammatory response in sepsis (Caruso et al., 2014). The TNF signaling pathway, known for its role in inflammation and immune regulation, could be affected by α-HB, potentially altering cytokine production and inflammatory responses (Wallaeys et al., 2024). The Toll-like receptor signaling pathway is essential for recognizing pathogen-associated molecular patterns and initiating immune responses, and α-HB’s influence on this pathway could impact immune cell activation and cytokine production (Glaser and Speer, 2013). We speculate that α-HB may modulate these pathways by interacting with specific receptors or signaling molecules involved in these pathways, thereby influencing the downstream signaling cascades and ultimately affecting the immune response and inflammation in sepsis. In addition, pathway activity evaluation using GSVA elucidated differences in immune and inflammatory pathways between the two α-HB-related subtypes. The pronounced enrichment of pathways related to immune response and inflammation in α-HB subtype 2 suggests a more aggressive inflammatory phenotype. This is corroborated by Antcliffe et al., who noted certain sepsis phenotypes associated with heightened inflammatory responses and worse clinical outcomes (Antcliffe et al., 2024). Regulation of autophagosome assembly and responses to interleukins, particularly IL-6, IL-2, and IL-15, may be crucial in driving this inflammatory phenotype. Elevated levels of IL-6 have been linked to poor outcomes in sepsis patients, reinforcing the potential of targeting these pathways for therapeutic intervention (DeMerle et al., 2024).

The analysis of immune cell infiltration using ssGSEA provided additional insights into the distinct immunological landscapes of the two α-HB-related sepsis subtypes. The higher infiltration of dendritic cells, regulatory T cells, and natural killer cells in α-HB subtype 1 suggests a more balanced immune response, which may facilitate effective pathogen clearance and tissue repair. In contrast, the increased presence of neutrophils and eosinophils in α-HB subtype 2 points to a hyper-inflammatory state, associated with acute tissue damage and organ dysfunction (Kwok et al., 2023; Sharma et al., 2023). These findings highlight the potential for targeted immunotherapeutic strategies considering each subtype’s unique immune profiles, echoing recent studies advocating for personalized sepsis treatment approaches (Sweeney et al., 2018). Critically, the survival analysis depicted in Figure 7C reveals a significant disparity in outcomes between these subtypes, with α-HB Subtype 1 predominantly linked to non-survivors and α-HB Subtype 2 more frequent among survivors. This survival divergence aligns with the GSVA and immune cell infiltration results: subtype 1’s balanced immune response, characterized by regulatory cell infiltration (e.g., dendritic and regulatory T cells), may contribute to immune exhaustion or suboptimal pathogen clearance, exacerbating mortality risks (Jiang et al., 2012). Conversely, subtype 2’s hyper-inflammatory phenotype, underscored by neutrophil and eosinophil infiltration and enriched inflammatory pathways (e.g., IL-6 response via GSVA), correlates with improved survival, potentially due to a potent early inflammatory burst that accelerates pathogen elimination and recovery, despite risks of tissue damage (Shen et al., 2017). Collectively, these integrated findings emphasize that α-HB exposure modulates sepsis outcomes through distinct inflammatory and immune-balancing mechanisms, informing future stratification and therapy.

In our study, the binding affinities reported (−4.1 to −4.8 kcal/mol) are relatively modest, which may indicate weak interactions. However, it is important to note that even weak interactions can be biologically relevant in certain contexts. For instance, transient and low-affinity interactions often play crucial roles in signaling pathways and regulatory mechanisms (Schreiber and Keating, 2011). Furthermore, the cumulative effect of multiple weak interactions can have significant biological outcomes (Scheepers et al., 2020). Previous studies have shown that similar binding affinities can still lead to meaningful biological activities (Kantidze and Razin, 2020). We acknowledge the limitations of our binding affinity data and propose that future studies involve functional assays to validate the biological significance of these interactions.

Despite the significant findings of this study, several limitations must be acknowledged. Importantly, our study design limits our ability to establish causality, as the correlational nature of the bioinformatics analysis cannot definitively prove that α-HB directly causes the observed molecular changes in sepsis. The identified associations may be influenced by unmeasured confounders or represent downstream consequences rather than primary causal events. Firstly, due to constraints in data availability from the GEO database, our analysis was limited to the survival information of sepsis patients, and we could not incorporate other clinical parameters such as SOFA scores. This restricts the comprehensiveness of our clinical correlations. Secondly, the study relies entirely on computational predictions and bioinformatics analyses without experimental validation in biological systems. The molecular docking results, pathway enrichments, and proposed drug-target interactions are purely computational predictions that require rigorous experimental verification. The functional roles of all proposed targets remain to be tested through *in vitro* and *in vivo* experiments. The modest binding affinities observed represent theoretical predictions that need biochemical validation to confirm their biological relevance. Additionally, the generalizability of our findings is constrained by the reliance on specific datasets; different cohorts may exhibit varying results. Future studies should include comprehensive experimental validation including cell-based assays, animal models, and clinical studies and use larger, diverse patient cohorts to strengthen the translational potential of our findings. Future experimental studies should focus on dose-response relationships, temporal analyses, and mechanistic validation through targeted interventions to establish causal relationships between α-HB exposure and sepsis outcomes. This limits the ability to confirm the identified targets and pathways’ functional roles in sepsis. Additionally, the generalizability of our findings is constrained by the reliance on specific datasets; different cohorts may exhibit varying results. Future studies should include experimental validation and use larger, diverse patient cohorts to strengthen the translational potential of our findings. Finally, while we identified potential biomarkers and molecular subtypes, further research is necessary to establish their clinical utility and therapeutic relevance in sepsis management.

## Conclusion

In conclusion, our study provides compelling evidence for the role of α-HB in modulating sepsis progression through its influence on immune responses, oxidative stress, and cellular signaling pathways. The identification of robust biomarkers and distinct sepsis subtypes lays the groundwork for future research aimed at elucidating the complex interplay between metabolic byproducts and host responses in sepsis. As we advance towards a more personalized approach to sepsis management, the insights gained from this study may inform the development of targeted therapies that address the unique molecular characteristics of α-HB-related sepsis subtypes. Future investigations should focus on the functional validation of identified biomarkers and the exploration of therapeutic strategies that leverage the distinct immune profiles observed in these subtypes.

## Data Availability

The original contributions presented in the study are included in the article/Supplementary Material, further inquiries can be directed to the corresponding author.
