# Supplementary material for: The role of α-hydroxybutyrate in modulating sepsis progression: identification of key targets and biomarkers through multi-database data mining, machine learning, and unsupervised clustering
Source: Front Pharmacol. 2025 Sep 17;16:1615269. doi: 10.3389/fphar.2025.1615269 (PMC12484119; doi:10.3389/fphar.2025.1615269)
Supplement: Supplementary file 2 [file Supplementaryfile2.docx]

Supplementary figures


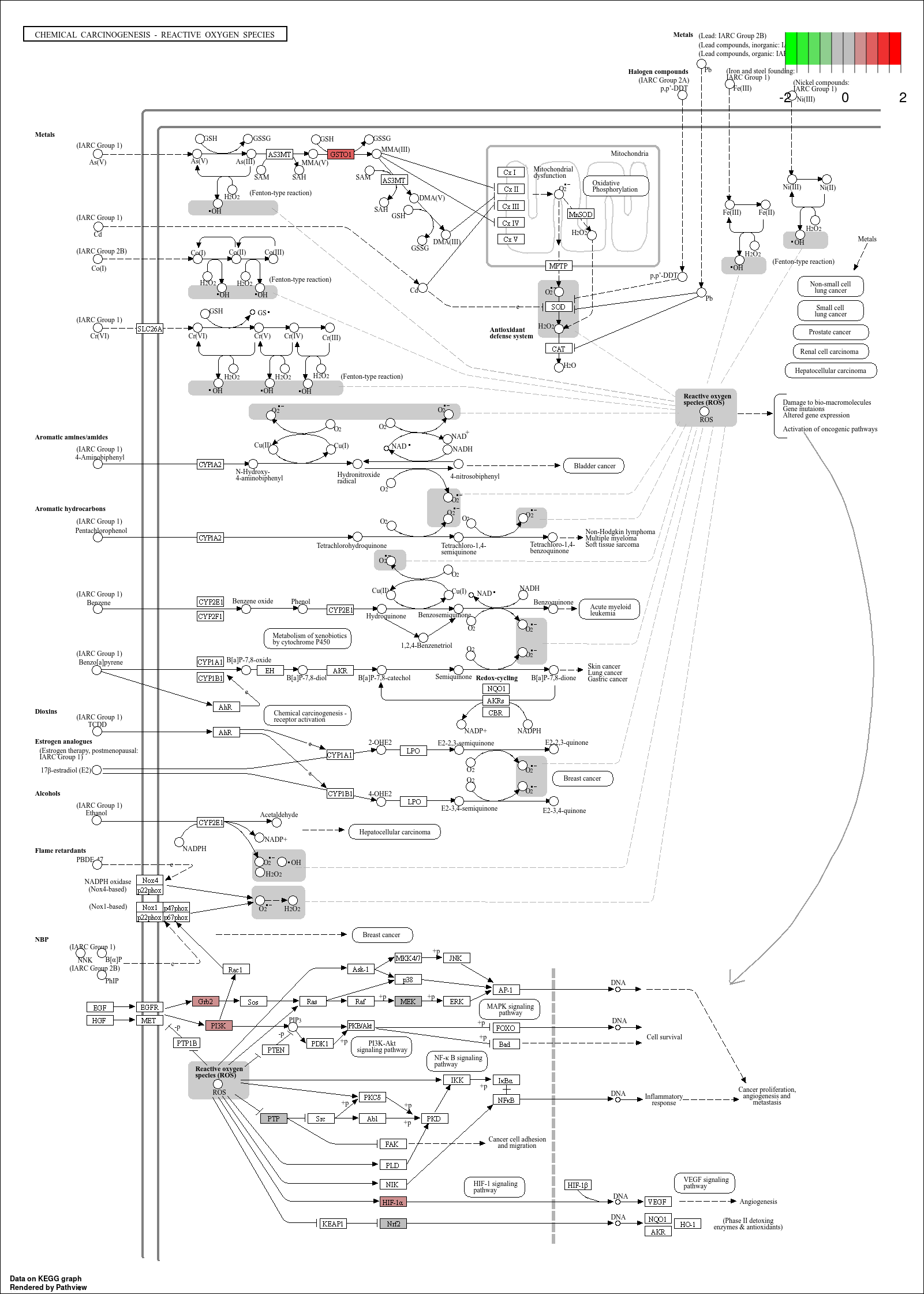


Figure S1 The toxicological mechanism of α-HB in sepsis involves the chemical carcinogenesis - reactive oxygen species pathway.


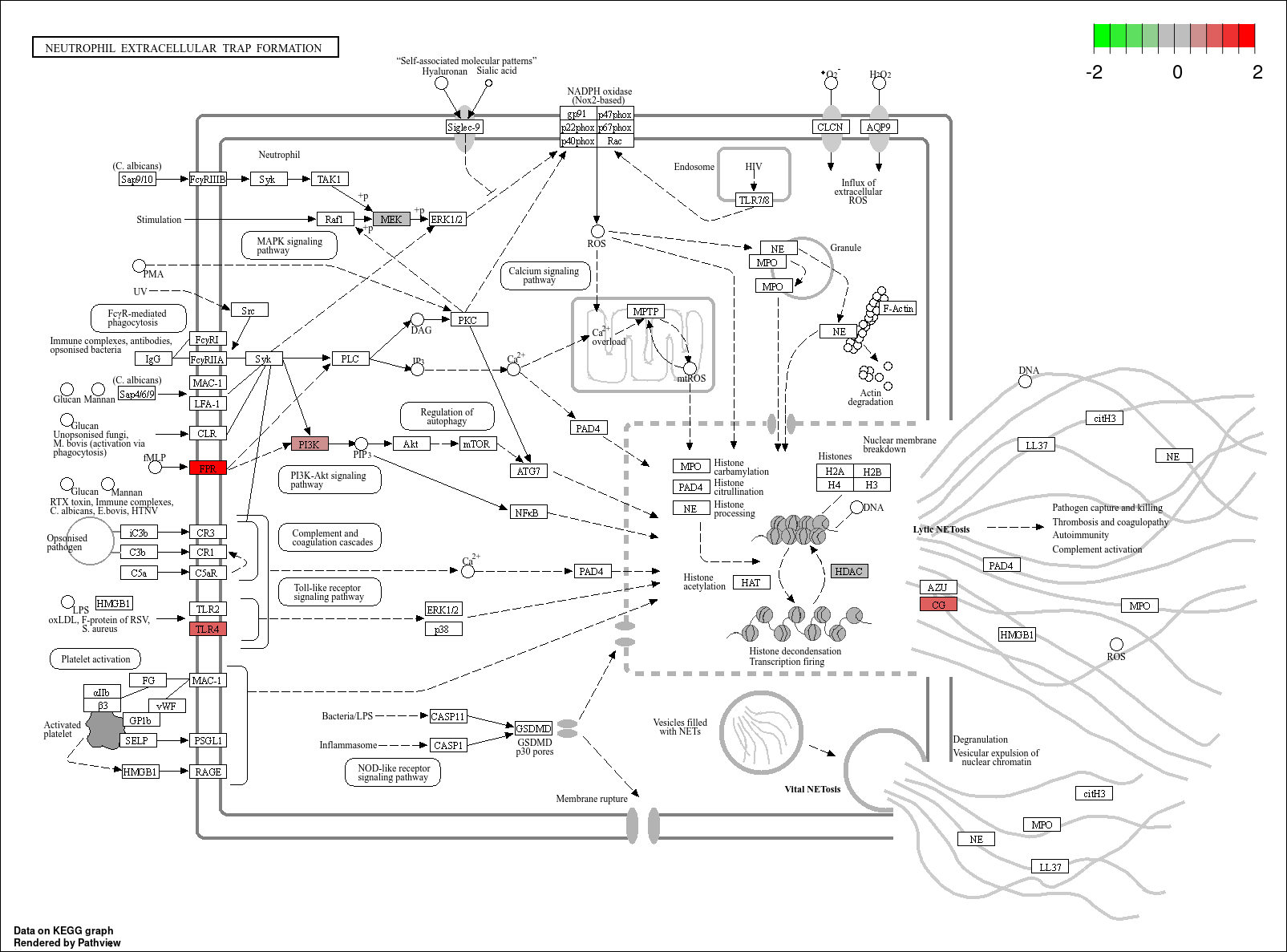


Figure S2 The toxicological mechanism of α-HB in sepsis involves the neutrophil extracellular trap formation.


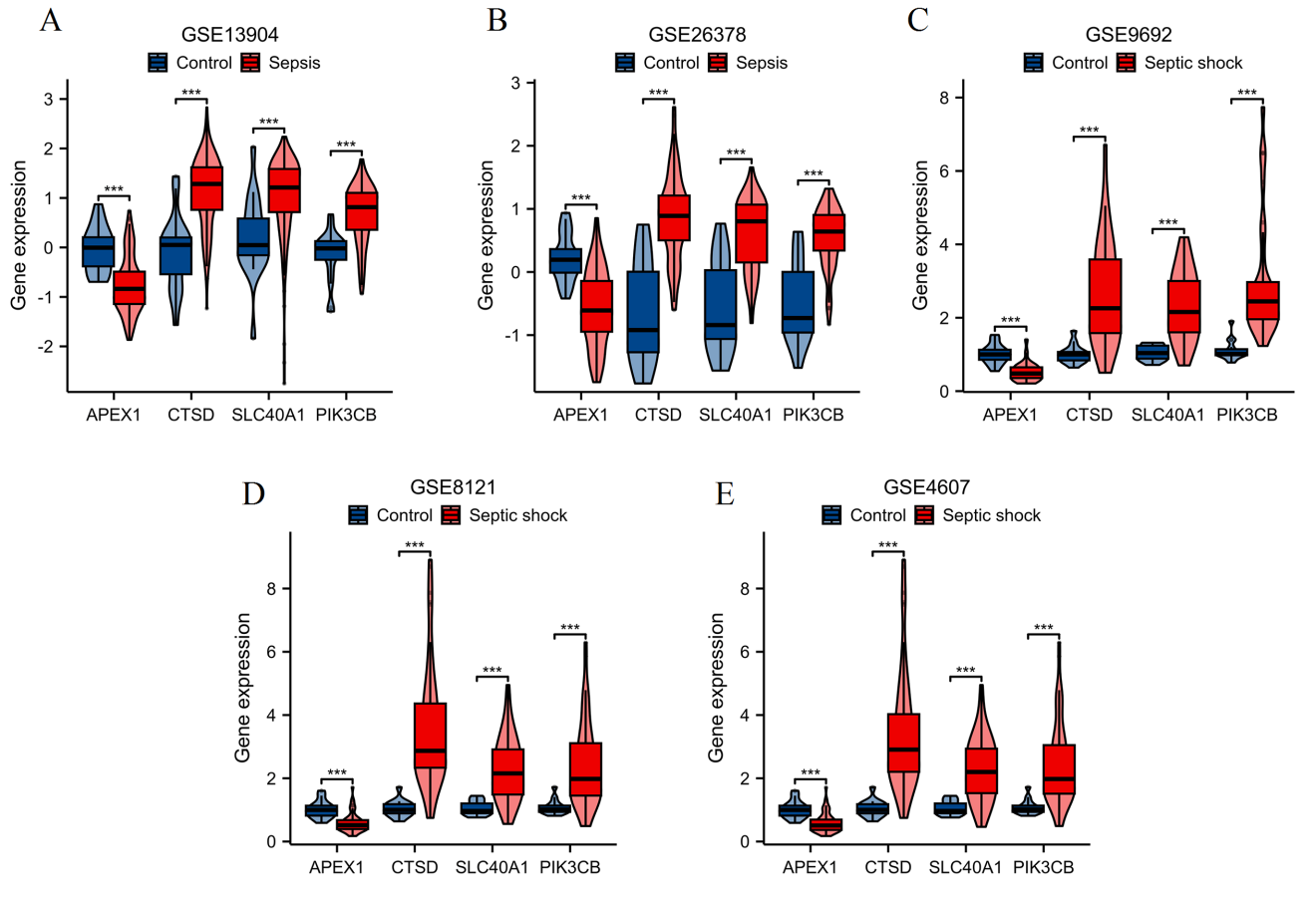


Figure S3 Validation of the four diagnostic genes in independent datasets GSE13904 (A), GSE26378 (B), GSE9692 (C), GSE8121 (D), and GSE4607 (E). ***p < 0.001.
